# 301. Immune Responses to Influenza Infection in Hospitalized Children Using Transcriptomics

**DOI:** 10.1093/ofid/ofad500.373

**Published:** 2023-11-27

**Authors:** Suchitra Rao, Kent Riemondy, Shaobing Li, Adriana Weinberg

**Affiliations:** University of Colorado School of Medicine, Aurora, Colorado; University of Colorado, Aurora, Colorado; University of Colorado School of Medicine, Aurora, Colorado; University of Colorado Denver, Denver, Colorado

## Abstract

**Background:**

Influenza infections produce a spectrum of disease severity in children. The host-response pathways associated with the progression to severe influenza disease and mechanisms of vaccination on mitigating illness severity are not well understood.

**Methods:**

We conducted a prospective study of children aged 2 to 18 years hospitalized with PCR-confirmed influenza infection from 2020-2022. We compared the host transcriptome using whole blood of children within 5 days of influenza infection (cases) with healthy asymptomatic controls undergoing elective surgery. We analyzed transcription profiles as a function of a) infected versus non-infected b) severe versus non severe and c) vaccinated versus unvaccinated. Pathway analysis on differentially expressed genes was performed.

**Results:**

Among 11 cases and 12 controls, there were no significant differences with respect to age, sex, or presence of a high-risk medical condition, but a higher proportion of cases were Hispanic (45.5% vs 8.3%, p = 0.0007) and unvaccinated (72.7% vs 33.3%, p = 0.036) (Table 1). Compared with healthy controls, 5,277 unique transcripts were differentially expressed in influenza-infected participants, of which 3,099 were up-regulated and 2,178 were down-regulated. Pathway analysis revealed gene enrichment related to interferon responses and innate inflammatory responses in cases (Figure 1). Further analyses comparing 6 severe with 5 moderate infection cases demonstrated 68 differentially expressed unique transcripts. We identified 102 differentially expressed genes in 3 cases with ≥1 dose of influenza vaccine compared to 8 unvaccinated, including 85 upregulated and 17 downregulated (Figure 2). B cell and neutrophil responses were upregulated, and innate inflammatory and interferon responses were downregulated in vaccinated cases (Figure 3). Moreover, vaccinated cases shared biosignatures with the uninfected controls.

**Figure:**
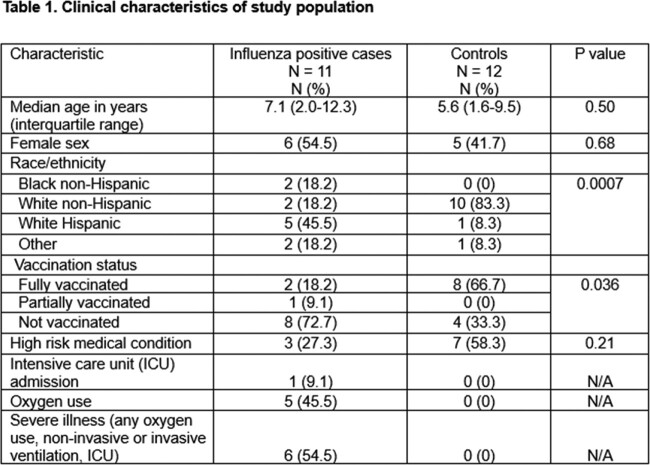

Heatmap of gene expression patterns of significant genes for influenza cases and controls.

**Figure:**
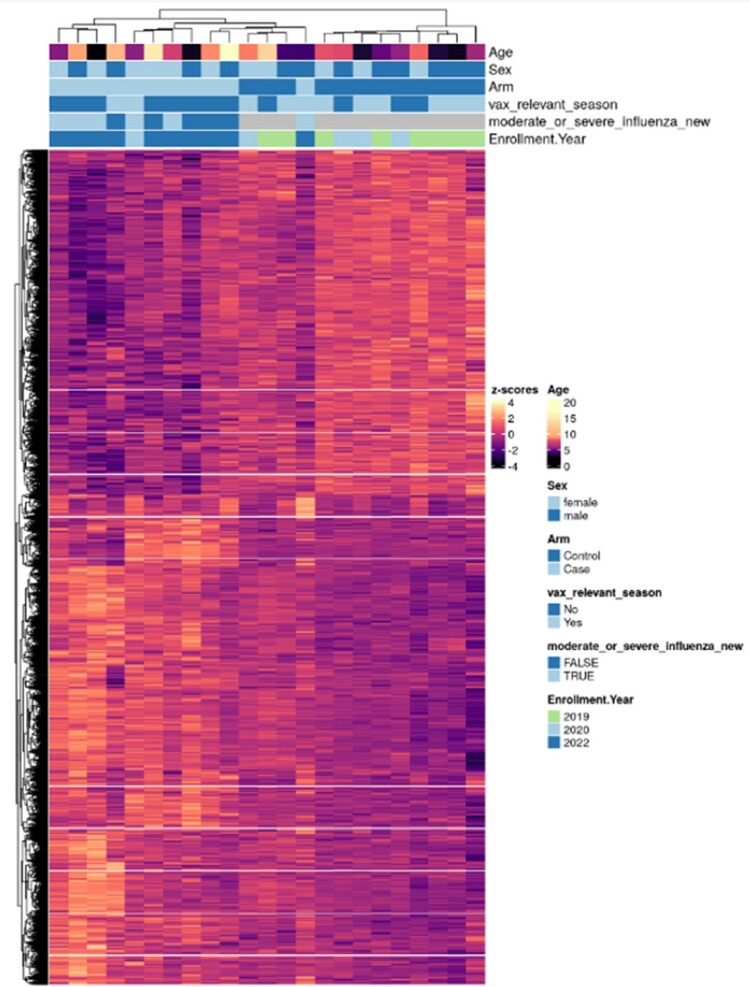

Legend: Heatmap showing the gene expression patterns of the significant genes across all samples. Centered and scaled normalized expression values are plotted. Highest upregulated genes compared to the average expression of that gene in all samples are light shades while darker colors represent downregulated genes.

Modular expression of immune responses in influenza cases and controls

**Figure:**
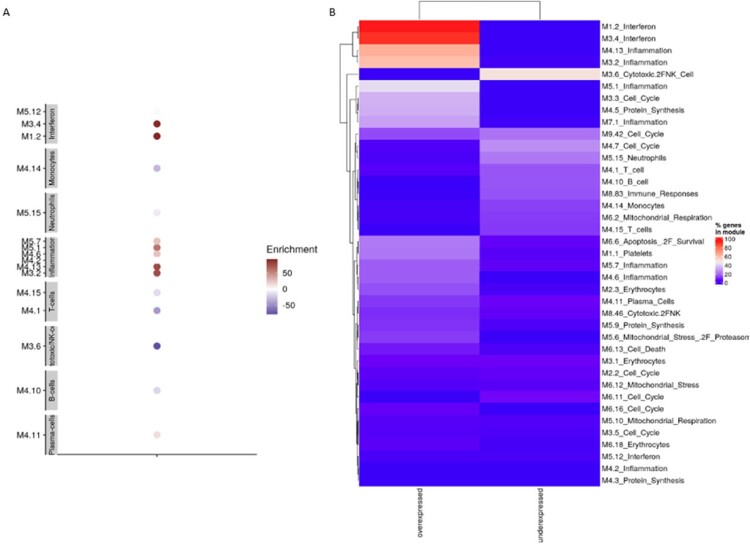

Legend: Panel A. Each module (M) represents a group of genes that are co-expressed and shared similar biological functions. Twenty-four selected modules pertaining to innate and adaptive immune responses are represented, including those related to interferons, monocytes, neutrophils, innate immunity/inflammation, T cells, cytotoxic/NK cells, plasma cells, B-cells, platelets, and undifferentiated cell cycles. The intensity of the color of the circles indicates the proportion of overexpressed (in red) or underexpressed (in blue) transcripts within each module (represented by a circle). Numeric values indicate the percentage of transcripts expressed in each specific module among influenza cases compared with controls. Panel B. Percentage of genes that are differentially expressed between influenza cases and controls. Left column represents genes that are overexpressed, right column represents genes that are underexpressed. Each row represents a module (M), summarizing a group of genes that are co-expressed and shared similar biological functions.

**Conclusion:**

Our study demonstrated important differences in inflammatory gene expression profiles of children with influenza infection compared with healthy controls, which were more pronounced in unvaccinated than vaccinated infected children. Our findings support disease mitigation through vaccination.

Modular expression in unvaccinated and vaccinated influenza cases

**Figure:**
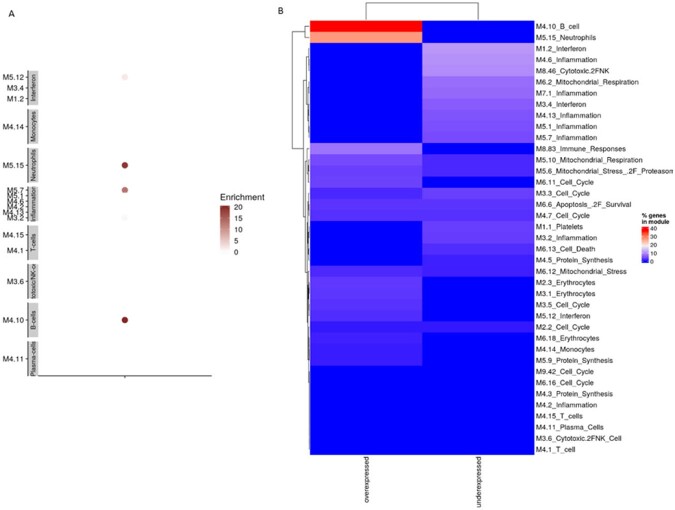

Legend: Panel A. Each module (M) represents a group of genes that are co-expressed and shared similar biological functions. Twenty-four selected modules pertaining to innate and adaptive immune responses are represented, including those related to interferons, monocytes, neutrophils, innate immunity/inflammation, T cells, cytotoxic/NK cells, plasma cells, B-cells, platelets, and undifferentiated cell cycles. The intensity of the color of the circles indicates the proportion of overexpressed (in red) or underexpressed (in blue) transcripts within each module (represented by a circle). Numeric values indicate the percentage of transcripts expressed in each specific module among influenza vaccinated cases compared with influenza unvaccinated cases. Panel B. Percentage of genes that are differentially expressed between vaccinated and unvaccinated cases. Left column represents genes that are overexpressed in vaccinated versus unvaccinated children, right column represents genes that are underexpressed in vaccinated versus unvaccinated children. Each row represents a module (M), summarizing a group of genes that are co-expressed and shared similar biological functions.

**Disclosures:**

**Suchitra Rao, MBBS, MSCS**, Sequiris: Advisor/Consultant **Adriana Weinberg, MD**, GlaxoSmithKline: Advisor/Consultant|GlaxoSmithKline: Grant/Research Support|Merck: Advisor/Consultant|Merck: Grant/Research Support|Seqirus: Advisor/Consultant

